# PARP inhibition radiosensitizes BRCA1 wildtype and mutated breast cancer to proton therapy

**DOI:** 10.1038/s41598-024-81914-w

**Published:** 2024-12-28

**Authors:** Mariam Ben Kacem, Scott J. Bright, Emma Moran, David B. Flint, David K. J. Martinus, Broderick X. Turner, Ilsa Qureshi, Rishab Kolachina, Mandira Manandhar, Poliana C. Marinello, Simona F. Shaitelman, Gabriel O. Sawakuchi

**Affiliations:** 1 Division of Radiation Oncology, Department of Radiation Physics, The University of Texas MD Anderson Cancer Center, Houston, TX USA; 2 Department of Biosciences, Rice University, Houston, TX USA; 3 Division of Radiation Oncology, Department of Breast Radiation Oncology, The University of Texas MD Anderson Cancer Center, Houston, TX USA; 4 Present Address: Department of Immunology, The University of Texas MD Anderson Cancer Center, Houston, TX USA; 5 Present Address: Department of Chemistry, Emory University, Atlanta, GA USA

**Keywords:** DNA repair inhibitor, PARP inhibitor, Radiosensitization, Proton therapy, Radiobiology, Radiotherapy, Breast cancer, Radiotherapy, Targeted therapies, DNA damage and repair, DNA damage response, Double-strand DNA breaks

## Abstract

Aggressive breast cancers often fail or acquire resistance to radiotherapy. To develop new strategies to improve the outcome of aggressive breast cancer patients, we studied how PARP inhibition radiosensitizes breast cancer models to proton therapy, which is a radiotherapy modality that generates more DNA damage in the tumor than standard radiotherapy using photons. Two human BRCA1-mutated breast cancer cell lines and their isogenic BRCA1-recovered pairs were treated with a PARP inhibitor and irradiated with photons or protons. Protons (9.9 and 3.85 keV/µm) induced higher cell kill independent of BRCA1 status. PARP inhibition amplified the cell kill effect to both photons and protons (9.9 and 3.85 keV/µm) independent of BRCA1 status. Numbers of γH2AX foci, micronuclei, and cGAS-positive micronuclei were significantly higher in BRCA1-mutated cells. Cell cycle distribution and stress-induced senescence were not affected by PARP inhibition in our cell lines. In vivo, the combination of protons (3.99 keV/µm) and PARP inhibition induced the greatest tumor growth delay and the highest survival. We found that PARP inhibition increases radiosensitization independent of BRCA1 status for both protons and photons. The combination of protons and PARP inhibition was the most effective in decreasing clonogenic cell survival, increasing DNA damage, and delaying tumor growth.

**Supplementary Information:**

The online version contains supplementary material available at 10.1038/s41598-024-81914-w.

## Introduction

Poly(ADP-ribose) polymerases (PARPs) are a family of proteins involved in several cellular processes. PARP1 is the most important member of the PARP family because of its involvement in DNA damage response (DDR) and DNA repair. The catalytic activity of PARP1 results in the recruitment of several proteins involved in DDR. PARP1 is actively involved in base excision repair, which repairs base damage and single-strand break (SSB) lesions^1^. PARP1 is also involved in sensing double-strand break (DSB) lesions^1^. In homologous recombination (HR), it helps to recruit BRCA1^2^. However, PARP1 has also been implicated in reducing HR by PARylation of BRCA1, which stabilizes BRCA1’s interaction with receptor-associated protein 80 (RAP80), limiting its function^3^. It is likely that PARP1 helps to “fine-tune” the level of HR^1,3^. PARP1 also has roles in non-homologous end joining (NHEJ), where it promotes the kinase activity of the DNA-dependent protein kinase catalytic subunit^4^. PARP1 has also been linked to alternative-NHEJ repair^1^. PARP1 is best known for its clinical relevance in BRCA1-mutant cancers. The loss of functional BRCA1 leads to a synthetic lethal relationship with PARP1 inhibition. The mechanistic basis for this relationship is thought to lie in the accumulation of SSB lesions and base damage that ordinarily would be repaired by PARP1 activity. During replication, these lesions become DSB lesions that generally do not create suitable substrates for NHEJ machinery and therefore require HR and functional BRCA1 activity to maintain cell viability.

PARP inhibition has been used as monotherapy in the treatment of BRCA1- or BRCA2-mutant breast cancers. Several studies have shown that BRCA1-mutated breast cancer cells are more sensitive to the cytotoxic effect of PARP inhibition than those that are BRCA1-intact^5,6^. Clinically, PARP inhibitors have improved outcomes compared with conventional therapy for both advanced and early-stage BRCA1/2-mutated breast cancer as well as ovarian and prostate cancers by extending disease-free and overall survival^7^. Several PARP inhibitors have been approved by both the US Food and Drug Administration and the European Medicines Agency to treat patients with BRCA1/2-deficient ovarian, breast, and castration-resistant prostate cancers. Of note, most of the current clinical approved PARP inhibitors also inhibit PARP2. Despite the observed improvement in outcomes from PARP inhibitors, up to 40% of patients with BRCA1/2-mutant cancers either do not respond initially to PARP inhibition or develop resistance to it^8^. Combinations of PARP inhibition with other agents have been proposed to overcome resistance in such patients^9–11^. The diverse role of PARP1 in DDR and DNA repair has raised interest in combining PARP inhibitors with other DNA-damaging therapies for patients who do not carry BRCA1/2 mutations. Radiotherapy is a particularly relevant option, because recent advancements in radiation delivery now allow the administration of high radiation doses to the tumor with relatively low doses to adjacent normal tissues, which may maximize the effect of PARP inhibition in the tumor while minimizing toxicity in normal tissue. Another advancement in radiotherapy has been the development of new treatment modalities such as proton therapy. In preclinical studies, protons were found to be more effective than photons for killing cancer cells because of their higher linear energy transfer (LET)^12^, which induces more complex and clustered DNA damage than do lower LET photons^12^.

PARP inhibitors have been observed to radiosensitize lung, melanoma, pancreas, liver, prostate, and breast tumors to photon radiation^13–18^. However, relatively little is known about how PARP inhibition affects cells treated with protons^19–21^. Radiotherapy is also known to enhance the formation of micronuclei (MN); cytoplasmic DNA, which often occurs in the form of MN, was recently recognized as a mediator of DDR–associated immune response through the cyclic GMP-AMP synthase (cGAS)-stimulator of interferon genes (STING) pathway^22^. This effect and others such as the induction of senescence^23,24^, which can have both tumor-suppressing and tumor-promoting effects owing to immunogenic effects^25–27^, are being recognized as important responses for further improving the effectiveness of radiotherapy.

Here we investigate PARP inhibition combined with clinical photon or proton beams in the context of BRCA1 wild-type and mutant breast cancer models. Specifically, we investigated BRCA1-mutant cell lines in tandem with isogenic pairs in which wild-type BRCA1 was restored in an aggressive breast cancer model. We found that the combination of protons plus PARP inhibition had the most effective antitumor effects in vitro and in vivo.

## Results

### PARP inhibition radiosensitizes wildtype and BRCA1 mutated breast cancer cells to photons and protons

**Fig. 1:**
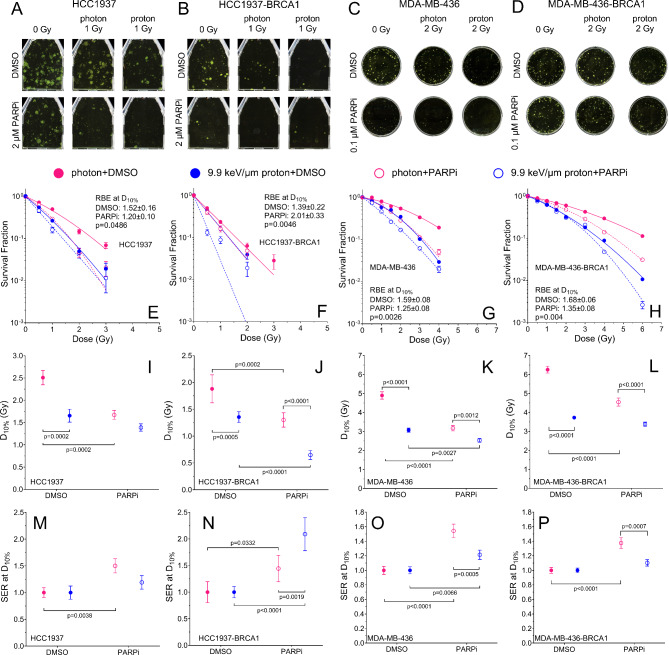
Clonogenic assays of two isogenic pairs of cell lines (HCC1937 and HCC1937-BRCA1; MDA-MB-436 and MDA-MB-436-BRCA1) after photon (6 MV x-rays) or proton (9.9 keV/μm) irradiation with DMSO or PARPi (2 µM for HCC1937 and HCC1937-BRCA1; and 0.1 µM for MDA-MB-436 and MDA-MB-436-BRCA1). (**A**–**D**) Representative colonies for (**A**) HCC1937, (**B**) HCC1937-BRCA1, (**C**) MDA-MB-436, and (**D**) MDA-MB-436-BRCA1 cells. (**E**–**H**) Survival curves and RBE at D_10%_. (**I**–**L**) D_10%_. (**M**–**P**) Sensitization enhancement ratio (SER) at D_10%_. Solid and dashed lines in (**E**–**H**) represent fits of the data using the linear quadratic model in which the α, β, and α/β ratio values are presented in Table S4, Table S5 and Table S6. Error bars represent the standard deviation. Each curve and bar represent the means of at least three independent experiments. One-way ANOVA with Tukey post hoc test was used to estimate p values.

HCC1937-BRCA1 and MDA-MB-436-BRCA1 were resistant to PARP inhibition compared to their BRCA1-mutated counterparts HCC1937 and MDA-MB-436, respectively (Fig. S1), confirming their BRCA1 restored phenotype^28,29^. Across all four cell lines, the survival fraction was lower after protons than after photons (Fig. 1A–H). All four cell lines were radiosensitized when PARP inhibitor (PARPi) was added, as quantified by the dose at 10% survival fraction (D_10%_) and sensitization enhancement ratio (SER) at D_10%_, except for the HCC1937 (BRCA1 mutated) and MDA-MB-436-BRCA1 (BRCA1 restored) cell lines exposed to protons (Fig. 1I–P, Table S1). The SER at D_10%_ was the highest in the photons + PARPi condition for HCC1937, MDA-MB-436 (BRCA1 mutated) and MDA-MB-436-BRCA1 cells and in protons + PARPi for HCC1937-BRCA1 (BRCA1 restored) (Fig. 1M–P, Table S2). The relative biological effectiveness (RBE) at D_10%_ was significantly lower with PARP inhibitor than with DMSO control, except for HCC1937-BRCA1 (Fig. 1E–H, Table S3).

### Persistent radiation-induced γH2AX foci increases with protons more than photons but is independent of PARP inhibition

**Fig. 2:**
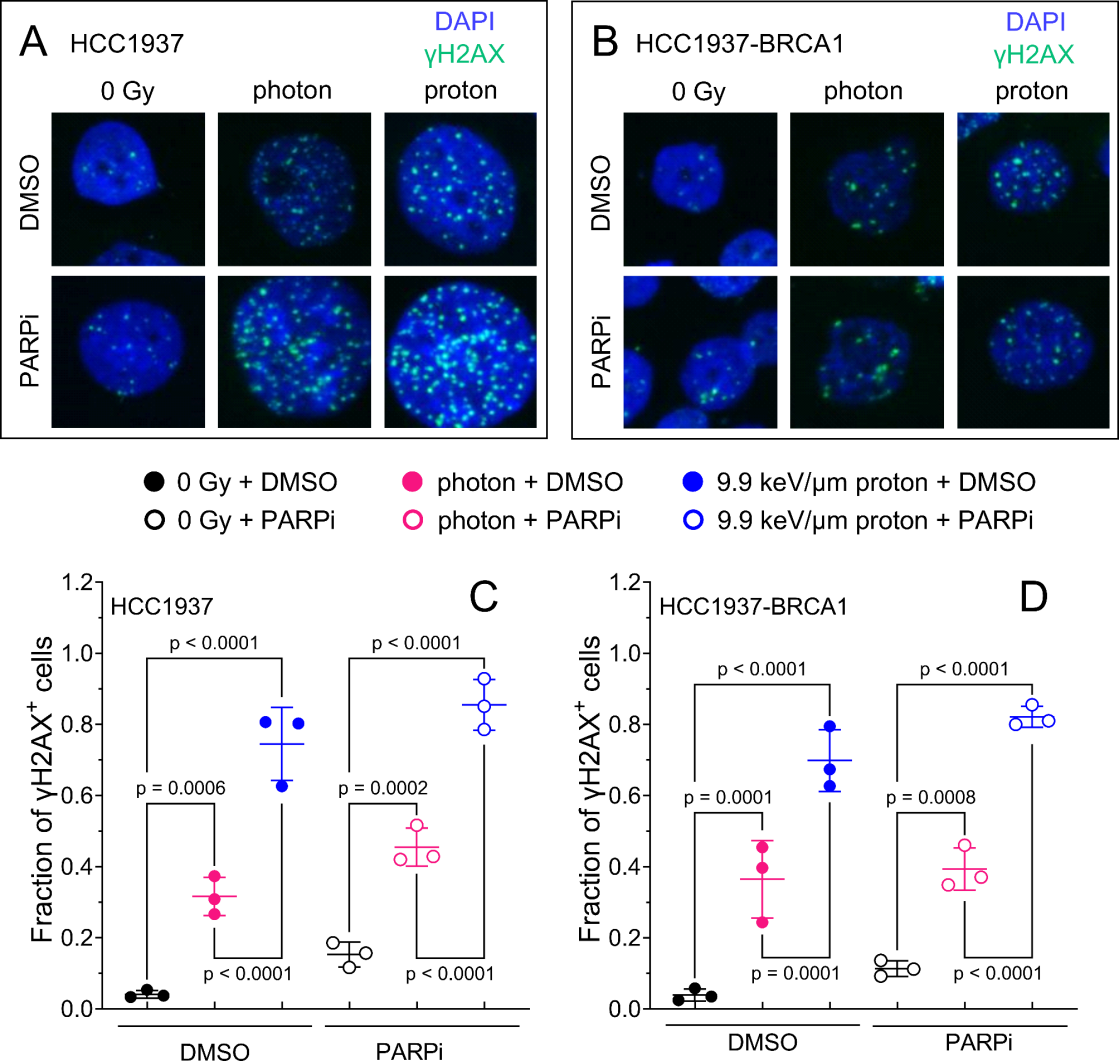
γH2AX foci at 72 h for BRCA1 mutated (HCC1937) and BRCA1-restored (HCC1937-BRCA1) breast cancer cell lines. Cells were irradiated with 5 Gy photons or protons or not irradiated (0 Gy) and treated with 2 µM of PARPi. (**A**,**B**) Representative images of γH2Ax foci. (**C**,**D**) Percentages of γH2Ax-foci positive cells in different treatment conditions for HCC1937 (**C**) and HCC1937-BRCA1 (**D**). Error bars represent the standard deviation. One-way ANOVA with Tukey post hoc test was used to estimate p values.

To understand how PARP inhibition and radiation influence residual DNA damage, we quantified γH2AX foci at 72 h after irradiation (5 Gy) and PARP inhibition (2 µM). We observed significant increases in γH2AX-foci positive cells in both HCC1937 and HCC1937-BRCA1 cells (Fig. 2A–D), with protons causing a larger increase than photons. The number of γH2AX-foci positive cells had a non-significant increasing trend after photons + PARPi or protons + PARPi in HCC1937 (Fig. 2C) and HCC1937-BRCA1 cells (Fig. 2D) compared with photons or protons alone.

### PARP inhibition does not modulate radiation-induced cell cycle arrest in HCC1937 or HCC1937-BRCA1 cells

**Fig. 3:**
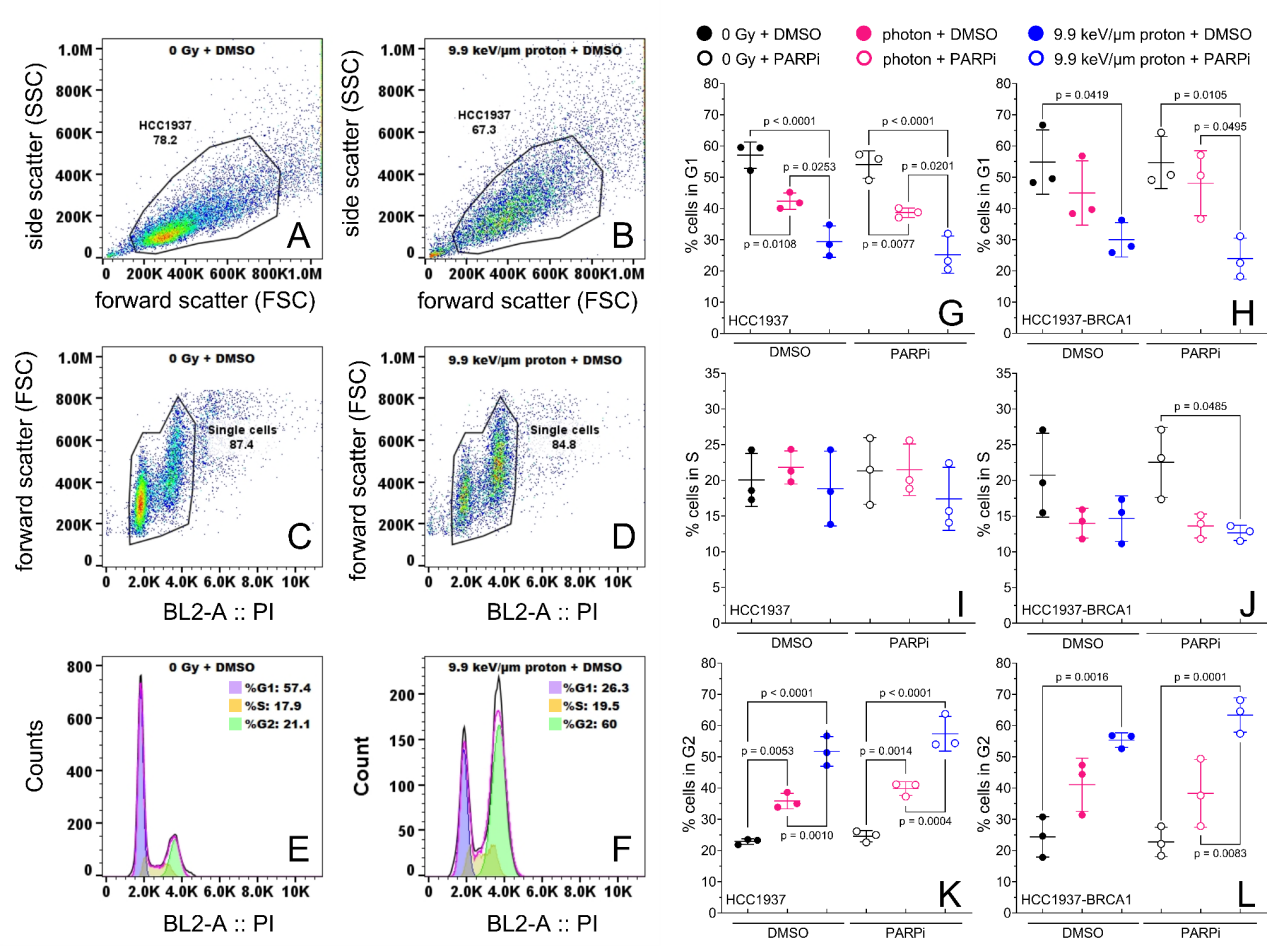
Cell cycle distribution at 48 h after treatment for BRCA1 mutated (HCC1937) and BRCA1-restored (HCC1937-BRCA) breast cancer cell lines. Cells were irradiated with 5 Gy photons or protons or not irradiated (0 Gy) and treated with 2 µM of PARPi. (**A**–**F**) Gating and quantification of cell proportion in different cell cycle phase for HCC1937. (**G**,**H**) Proportions of cells in G1 phase; (**I**,**J**) in S phase; and (**K**,**L**) in G2 phase. Error bars represent standard deviation. One-way ANOVA with Tukey post hoc test was used to estimate p values.

We next assessed the distribution of cells in G1, S, and G2 phases after photons or protons (5 Gy) combined with PARP inhibition (2 µM) (Fig. 3). For HCC1937 and HCC1937-BRCA1 cells, the proportion of cells in G1 decreased after both types of radiation with a significantly greater decrease after protons for HCC1937 but PARP inhibition did not modulate G1 proportion (Fig. 3G,H). The proportion of cells in S phase remained the same for all treatment conditions for HCC1937 cells and was decreased in HCC1937-BRCA1 cells after irradiation but PARP inhibition did not modulate S proportion (Fig. 3I,J). For HCC1937 and HCC1937-BRCA1 cells, the proportion of cells in G2 increased after both types of radiation, with protons presenting a higher increase than photons but PARP inhibition did not modulate G2 proportion (Fig. 3K,L).

### PARP inhibition does not modulate radiation-induced micronuclei nor radiation-induced cGAS colocalized micronuclei in HCC1937 or HCC1937-BRCA1 cells

**Fig. 4:**
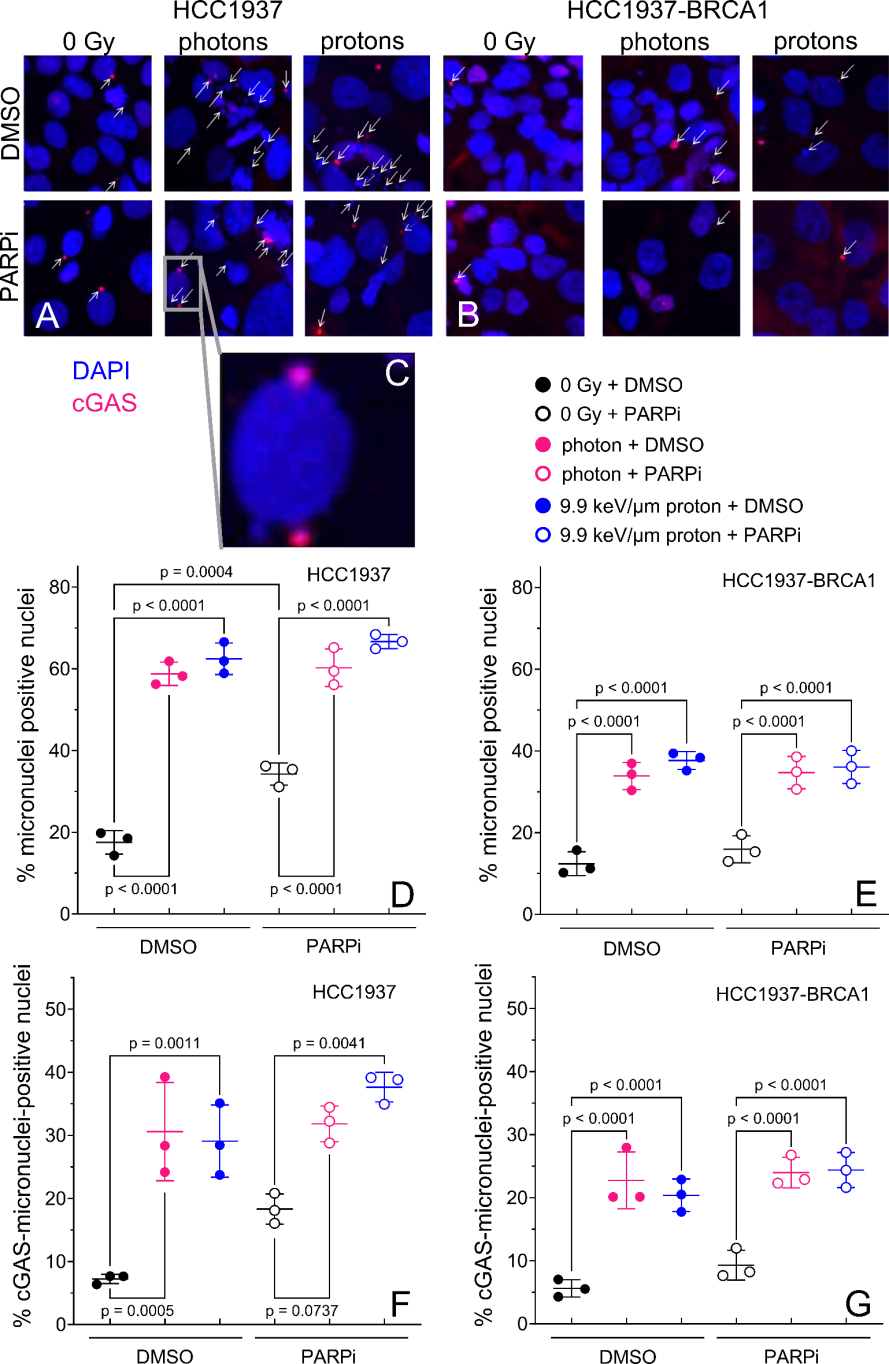
Micronuclei and cGAS colocalized micronuclei at 72 h after irradiation. (**A**–**C**) Representative images of micronuclei and cGAS colocalized micronuclei. Arrows point to MN with or without cGAS colocalization. (**D**,**F**) HCC1937 cells and (**E**,**G**) HCC1937-BRCA1 cells were irradiated with 5 Gy of photons or protons or not irradiated (0 Gy) and treated with 2 µM of PARPi. Error bars represent standard deviation. One-way ANOVA with Tukey post hoc test was used to estimate p values.

PARP inhibition alone increased MN proportion and cGAS^+^ MN proportion in HCC1937 cells but not in HCC1937-BRCA1 cells (Fig. 4). MN proportion was significantly higher in irradiated (5 Gy) cells than in unirradiated cells, but PARP inhibition (2 µM) did not modulate MN proportion after radiation (Fig. 4D,E). MN-cGAS-positive nuclei proportion increased after radiation but PARP inhibition did not modulate MN-cGAS-positive nuclei proportion after radiation (Fig. 4F,G). After both types of radiation, the MN proportion and MN-cGAS-positive nuclei proportion were higher for HCC1937 than for HCC1937-BRCA1 cells (Fig. S2).

### PARP inhibition does not modulate radiation-induced senescence in HCC1937 or HCC1937-BRCA1 cells

**Fig. 5:**
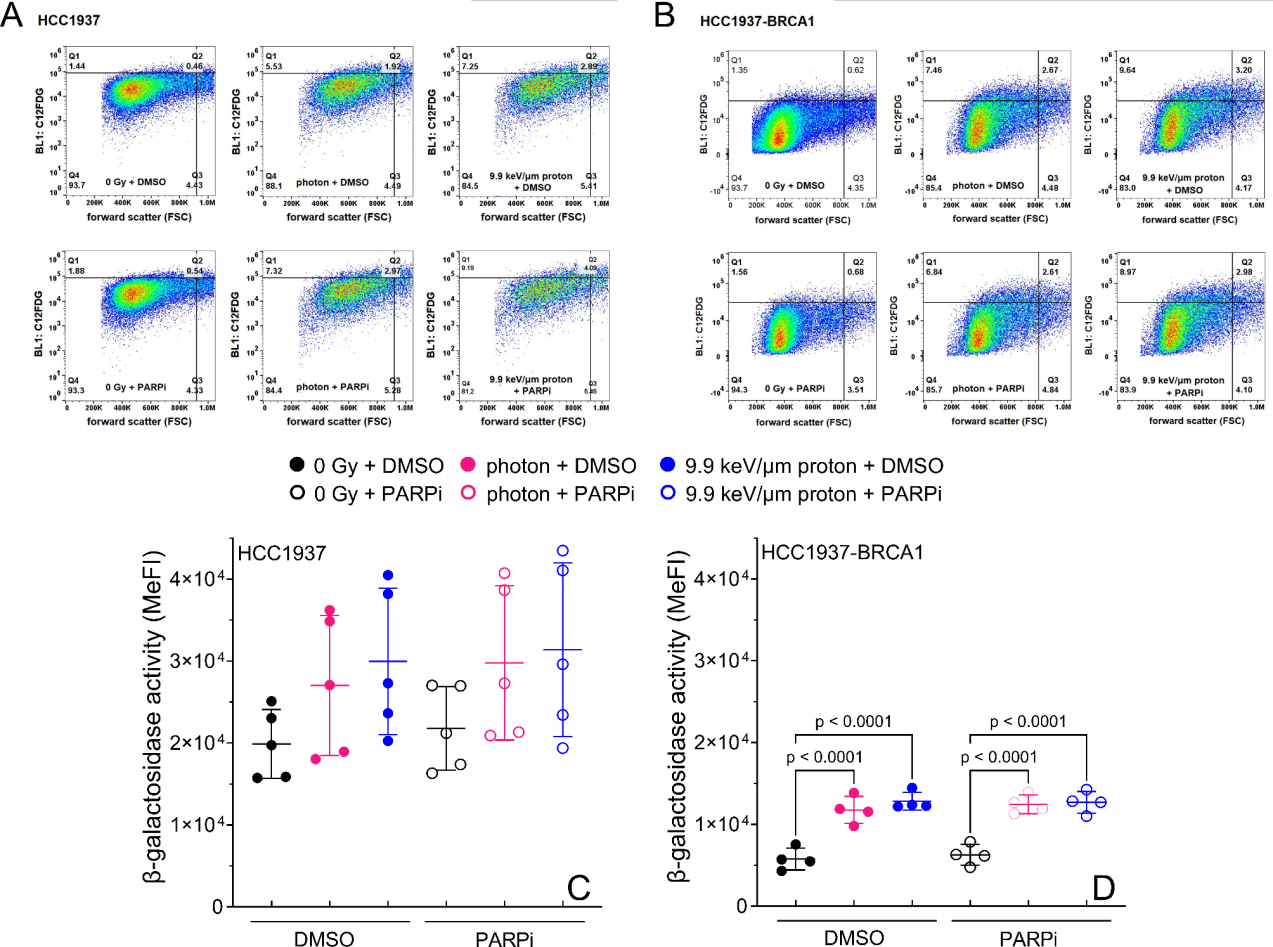
Flow-cytometric evaluation of radiation-induced senescence (C_12_FDG) at 7 days after radiation. (**A**,**B**) Senescence (cytometry panel). Representative flow cytometry measurements at 7 days after irradiation. Each bi-parametric representation (Size (FSC)/C12FDG (BL-1)) represents one independent experiment for at least 5 × 10^4^ living cells. (**B**). HCC1937 (BRCA1-mutated) and HCC1937-BRCA1 (BRCA1-recovered) cells were treated with 5 Gy of photons or protons or not irradiated (0 Gy) and treated with 2 µM of PARPi. (**C**,**D**) The mean intensity of β-galactosidase activity (associated with stress-induced senescence) is shown. Error bars represent standard deviation. MeFI, mean fluorescence intensity. One-way ANOVA with Tukey post hoc test was used to estimate p values.

For HCC1937, neither radiation (5 Gy) nor PARP inhibition (2 µM) affected β-galactosidase activity levels, a marker of senescence^30^ (Fig. 5A,C). For HCC1937-BRCA1 cells, β-galactosidase activity levels increased significantly after radiation but PARP inhibition did not modulate β-galactosidase activity levels (Fig. 5B,D). When comparing the two cell lines, in all conditions, HCC1937 had significantly higher β-galactosidase activity than did HCC1937-BRCA1. At baseline (no radiation), β-galactosidase activity was significantly higher for HCC1937 than for HCC1937-BRCA1 cells (Fig. S3).

### Protons combined with PARP inhibition significantly delay tumor growth

**Fig. 6:**
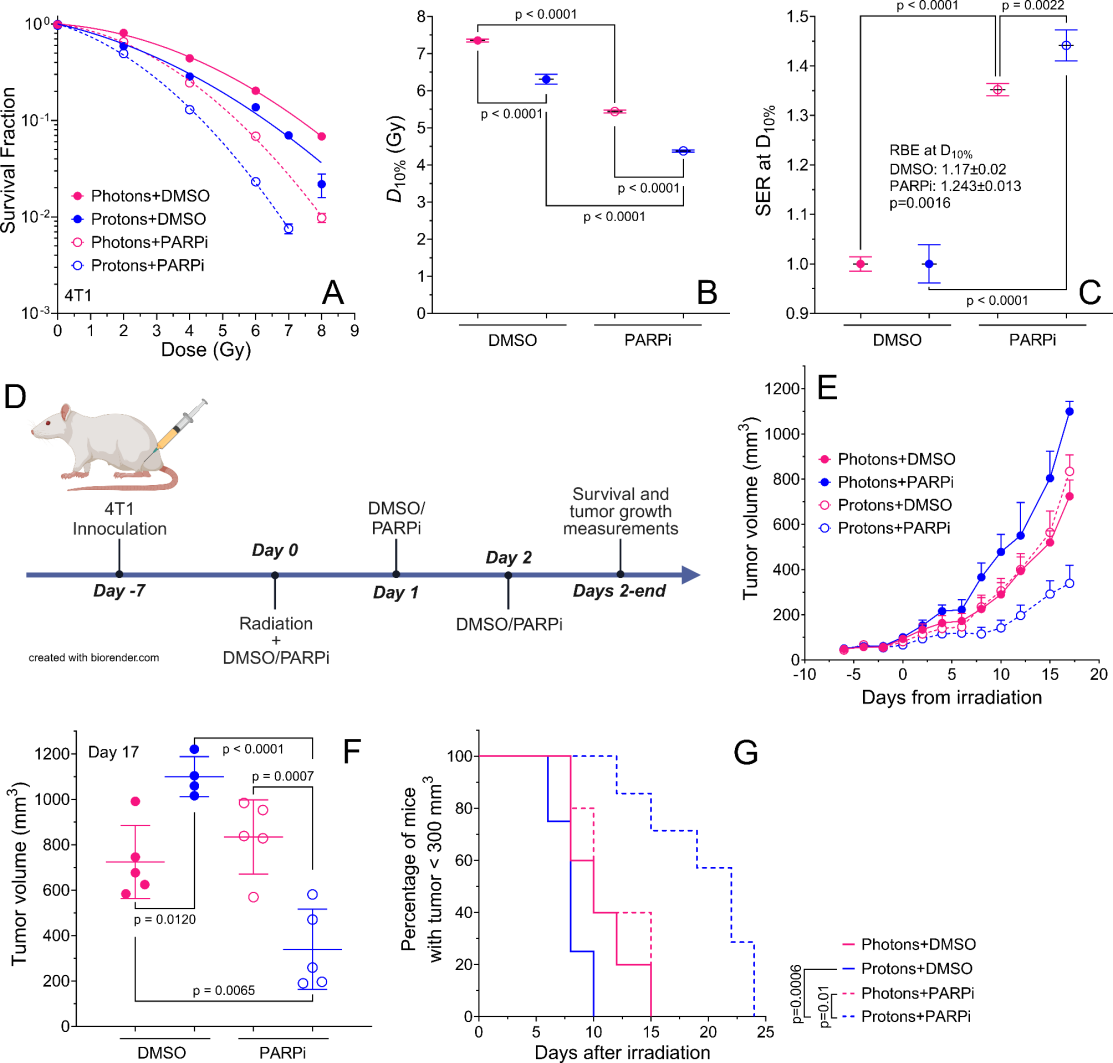
4T1 tumor model. (**A**–**C**) Clonogenic assay for 4T1 cells after photons (6 MV x-rays) or protons (4 cm SOBP, 3.85 keV/µm) with DMSO or PARPi (1 µM). Each curve and data point represent the means of at least three independent experiments. (**A**) Survival fraction, (**B**) D_10%_, (**C**) SER at D_10%_ and RBE at D_10%_. (**D**) Animal experiment plan. (**E**–**G**) Tumor volume analyses in a 4T1 tumor model treated with photons or protons plus PARPi or DMSO. (**E**) Tumor growth delay over time after radiation. (**F**) Tumor volume at day 17; and (**G**) percentage of mice with tumor volumes that reached 300 mm^3^. Mice were exposed to a single 11-Gy dose of photons or protons on day 0. PARPi was administrated during three consecutive days at a dose of 100 mg/kg per day starting 2 h before irradiation. Vehicle indicates DMSO. One-way ANOVA with Tukey post hoc test was used to estimate p values in (**B**,**C**,**E** and **F**). The log-rank test was used to estimate p values in (**G**). Error bars represent the standard deviation (**A**–**C**,**F**) or standard error of the mean (**E**).

We first assessed the response of 4T1, a murine BRCA1-proficient triple negative breast cancer cell line, in vitro by clonogenic survival assays. The clonogenic survival curves of 4T1 cells were lower for protons than photons (Fig. 6A). The D_10%_ was significantly decreased after protons compared to photons (Fig. 6B); the decrease was significantly greater when cells were treated with PARPi (1 µM). The 4T1 cell line is more radioresistant than the human cell lines used in this work (Table S1). The SER at D_10%_ for 4T1 was higher for protons compared to photons (Fig. 6C) and compared to the human cell lines, the SER at D_10%_ for both photons and protons presented intermediate values (Table S2). The RBE at D_10%_ was significantly higher with PARP inhibitor than with DMSO control. Compared to the human cell lines, the RBE at D_10%_ values (DMSO and PARPi) of 4T1 cells were generally lower (Table S3).

For tumor growth delay in the 4T1 tumor model, treatments with either photons or protons alone or with photons + PARPi or protons + PARPi had an initial effect in the animal weight but their weights were recovered after day 10 from start of treatment (Fig. S4). Protons alone was less effective than photons alone (Figs. 6D,E and S5). However, protons + PARPi demonstrated the highest relative delay in tumor growth compared with all other groups (Figs. 6D,E and S5). Mice treated with protons + PARPi had significantly smaller tumor volumes than did mice treated with photons + PARPi at day 17 after radiation (Fig. 6F), with an initial tumor volume of around 80 mm^3^ (Fig. S6). Kaplan–Meier curves indicating time for tumor volumes to reach 300 mm^3^ also showed significant delay in tumor growth for protons + PARPi compared with the other conditions (Fig. 6G). Overall mouse survival was non-significantly increased in mice treated with photons + PARPi or protons + PARPi compared to photons or protons alone (Fig. S7).

## Discussion

This study corroborates preclinical evidence that regardless of BRCA1 status, PARP inhibition radiosensitizes cancer cells including breast cancer cells across two distinct forms of radiotherapy, probably through unresolved DNA damage^13–15,18−21,31^.

We confirmed here that protons are more effective in killing breast cancer cells independent of BRCA1 status (Fig. 1). For some of the data, the LET of 9.9 keV/µm was greater than that achieved at the middle of the spread-out Brag peak (SOBP) (2–4 keV/µm), which probably explains why the RBE values at D_10%_ in our study were much greater than the clinically used 1.1 value. However, we did observe an RBE at D_10%_ of 1.17 for the 4T1 cell line for an LET of 3.85 keV/µm. BRCA1 is well known to operate in HR, where it facilitates loading RAD51 onto single-stranded DNA^32^. Defects in HR are thought to enhance sensitivity to high-LET radiation because of DNA damage clustering^33^, which NHEJ is ineffective to repair. Although the importance of HR increases with LET^34,35^, NHEJ remains by far the most important pathway in radiation response^35^. This may explain the lack of difference in RBE according to BRCA1 status.

PARP inhibition can lead to unresolved SSB lesions and bulky adducts at single-strand damage sites because of PARP1 trapping^36^. Both lesion types eventually require BRCA1-mediated HR for the cell to remain viable, which is unavailable in BRCA1-mutant cell lines, leading to cell death. PARP inhibition increased the radiosensitivity to both radiation types and generally reduced the RBE, except in HCC1937-BRCA1 cells. We believe that this finding results from a proportion of the damage induced by high-LET protons and relatively high dose for the radiosensitive HCC1937 isogenic cell lines being lethal without disrupting the DNA repair response, thereby conferring no added benefit from disruption of PARP1; however, a larger proportion of the damage induced by photons becomes lethal only if PARP1 is disrupted. Similarly, Park et al.^21^ observed unchanged RBE values of BRCA1-proficient breast cancer cell lines treated with Olaparib and protons compared to protons alone. Notably, however, even though the RBE was modestly reduced or unchanged, combining protons with PARP inhibition was the most effective treatment for cell killing, with the lowest D_10%_ observed in all cell lines tested owing to the higher LET of protons. This is an important consideration for clinical translation when different radiation modalities are combined with a radiosensitizing agent, and for considering whether a fixed RBE is the most appropriate metric when combining protons with radiation modulators.

In both BRCA1-mutant and wildtype cells, persistent γH2AX foci at 72 h after irradiation was significantly higher for 9.9 keV/µm protons alone compared to photons alone, indicating that proton-induced DNA damage is harder to repair, which could be due to the proton-induced DNA damage being more complex and clustered than that of photons. However, this needs to be further studied. In both BRCA1-mutant and wildtype cells, PARP inhibition (compared to DMSO) induced a non-significant increase in persistent γH2AX foci after photons or protons. The small increase in γH2AX foci after PARP inhibition could be related to the relatively high dose of 5 Gy used for the radiosensitive HCC1937 isogenic pair, in which a dose of 5 Gy may be sufficient to overwhelm the cells with DNA damage. Similarly to our data, Park et al.^21^ observed a trending of more γH2AX foci after combining radiation (either photons or protons) with Olaparib compared to radiation alone.

We further explored the response to photons and 9.9 keV/µm protons by investigating cell cycle distribution. An important aspect of a cell’s response to irradiation is temporarily halting cell cycle progression^37,38^. This allows the cells time to repair DNA damage, preventing mitotic cell death. Sensitivity arising from PARP inhibition is thought to be attributable in part to PARP1 trapping^36^, which creates lesions that prevent cell cycle progression because the DNA replication machinery cannot replicate through the chromatin-PARP1 complex. Ultimately, this can lead to stalled and eventually collapsed forks and cell death^39,40^. Our results suggest that the reduction in G1 populations in BRCA1-mutant cells was much more pronounced with radiation regardless of PARP inhibition. Although BRCA1-restored cells showed a significant decrease in G1 populations when PARP inhibition is combined with protons compared with photons (Fig. S8). This was accompanied by an increase in cells in G2. This suggests that transition to M is blocked for a prolonged period. Protons alone led to greater G1 depletion and G2 block than photons alone, probably because of the higher LET and increased persistent DNA damage as indicated by our γH2AX foci data.

A further cell cycle–related effect is the induction of senescence. Our data supports the concept that radiation-induced DNA damage triggers senescence^24^. However, in our BRCA1 mutated cells, β-galactosidase levels are not impacted by radiation type or PARP inhibition in our cell line. We hypothesized that protons alone would induce greater levels of senescence than photons alone because the higher LET of protons would result in more unresolved DNA damage, which we observed when we examined residual γH2AX foci and cell cycle. This finding has been linked with permanent blockade of the G1/S transition, which is also associated with increased senescence-associated β-galactosidase activity^41,42^. Prolonged G2 arrest has also been linked with radiation-induced senescence^43,44^, which agrees with our findings. However, the combination of PARP inhibition with radiation or radiation type (photons versus protons) seems to have no effect on senescence induction in our cell line. This is in contrast to Huart et al.^45^ who observed that radiation combined with Olaparib could increase senescence when lower doses of the inhibitor and radiation were used. In our study, no additional senescence induction was gained from combining radiation with Olaparib. This could be because the radiation dose we used may be sufficiently high to induce senescence on its own. Also, this could be a cell line dependent effect and needs to be confirmed in other cell lines. Moreover, a BRCA1 mutation has been associated with increased cellular reactive oxygen species^46–48^, which could predispose BRCA1-mutated cells to stress-induced damage, which could also explain the higher level of senescence in BRCA1 mutated cells. Indeed, in the absence of PARP inhibition, senescence was approximately three times higher in BRCA1-mutated cells than in BRCA1-recovered cells (Fig. S9). Radiation-induced senescence has been observed across a range of LET values^49,50^. Interestingly, Schniewind et al.^50^ showed that protons (3.7 keV/µm) led to lower levels of senescence compared with photons, possibly because of more cell death. This is in contrast with our data and needs to be further studied since many factors probably dictate whether a cell dies or enters senescence, including intrinsic factors like genetic abnormalities (e.g., p53 status), extrinsic factors such as radiation dose, tumor microenvironment, and modifying factors such as PARP inhibition.

Current findings suggest that a key signaling mechanism by which immune activation is achieved after radiotherapy is through cytoplasmic DNA fragments originating from ruptured MN, which activate the cGAS-STING pathway^51–53^. Our results showed that cells with mutant BRCA1 had more baseline MN than BRCA1-restored cells and that PARP inhibition alone could increase MN in BRCA1-mutant cells, probably because of the reduced HR capacity but also the increased requirement for HR owing to the unrepaired lesions induced by PARP inhibition and PARP1 trapping. We noted more cGAS-positive MN in BRCA1-mutant cells, and PARP inhibition could increase this. No differences in cGAS-positive MN were noted in BRCA1-restored cells when radiation was combined with PARP inhibition. Several factors could influence the binding of cGAS to DNA, such as the size of the DNA fragment and the presence of other proteins such as DNA repair proteins that could mask the cGAS-binding site in double-stranded DNA. Also, the lack of BRCA1 could lead to more binding sites for cGAS to bind to DNA because downstream HR proteins such as RPA and Rad51 are not recruited properly. Another consideration that may influence cGAS binding is the size of the DNA fragment, with longer DNA fragments being more effective at activating cGAS^54^. This effect is probably the most influenced by radiation type, because in a cell-free system, higher-LET radiation has been shown to lead to shorter DNA fragments^55,56^. However, we did not observe any less cGAS binding in the proton-irradiated groups relative to the photon-irradiated groups.

Finally, using an aggressive triple negative breast cancer model that is BRCA1 wildtype, we show that protons combined with PARP inhibition significantly delayed tumor growth (Fig. 6), supporting the in vitro studies indicating that the combination of protons and PARP inhibition generates more cell kill and persistent DNA damage than photons + PARPi, photons alone or protons alone. Protons alone presented a lower effectiveness to delay tumor growth than photons alone, which is contrary to our in vitro preliminary data. This could be due to difficulties to cover the entire tumor with protons with a homogeneous proton dose because the lateral penumbra of the proton beam is much sharper than that of the photon beam, which makes it challenging to align the tumors at the beam edge while sparing their bodies. The ratio of the tumor volume on day 17 between protons + PARPi vs. protons alone (3.2 ± 1.7, mean ± SD) is significantly higher compared to photons + PARPi vs. photons alone (0.87 ± 0.25, mean ± SD), indicating that PARPi radiosensitizes protons more than photons for 4T1 tumors.

Our studies have limitations. We used a high LET for protons (9.9 keV/µm) and a relatively high dose (5 Gy) for a radiosensitive cell line (HCC1937) for most of the in vitro assays. The high LET and relatively high dose for a radiosensitive cell line could have masked some of the effects of PARP inhibition. Clinically, LET regions of around 9.9 keV/µm is only present in the distal edge of the beam (residual range in water of about 0.20–0.24 mm) in very small regions of the tumor and whether these high LET values are clinically relevant need to be further studied. Another limitation is that although not significant, the tumor volume at the day treatments started (day 0) was systematically lower for the photons + PARPi and protons + PARPi groups compared to those for the photons alone and protons alone groups (Fig. S6). However, because the initial tumor volumes for photons + PARPi and protons + PARPi are very similar, our conclusion that at day 17 protons + PARPi is more effective than photons + PARPi still holds. We used a single dose of 11 Gy for our in vivo studies because of difficulties with proton beam time availability for consecutive days to enable a more clinically relevant fractionated schedule. However, we confirmed that the tumor growth delay of one fraction of 11 Gy is equivalent to a more clinically relevant fractionated schedule of 6 Gy per fraction in three consecutive days to a total of 18 Gy (Fig. S10).

In summary, our data indicate that PARP inhibition radiosensitizes both BRCA1-mutant and BRCA1-recovered breast cancer cells to photons and protons. The relative biological effectiveness is modulated by PARP inhibition. The combination of protons and PARP inhibition was the most effective in decreasing clonogenic cell survival, increasing DNA damage, and delaying tumor growth.

## Methods

### Cell lines

We used two human triple negative breast cancer cell lines with mutated BRCA1 (HCC1937 and MDA-MB-436) and their respective isogenic cell lines with restored BRCA1 (HCC1937-BRCA1 and MDA-MB-436-BRCA1). The MDA-MB-436 isogenic pair was obtained towards the conclusion of this project to serve as a secondary isogenic pair to confirm the clonogenic cell survival data for the HCC1937 isogenic pair. Of note, the HCC1937 is a challenging cell line to use for the clonogenic cell survival assay. Thus, the HCC1937 isogenic pair was used for all assays, while the MDA-MB-436 isogenic pair was only used for clonogenic cell survival. For in vivo studies, we used the BRCA1-intact murine triple negative breast cancer cell line 4T1. All cell lines were maintained in a humidified atmosphere at 37 °C, 5% CO_2_ in air. MDA-MB-436 and MDA-MB-436-BRCA1^28^ cells were kindly provided by Dr. N. Johnson (Fox Chase Cancer Center) and cultured in RPMI-1640 medium (10-040-CV, Corning, Glendale, AZ) with 10% fetal bovine serum (FBS) (F0926, Sigma Aldrich, St. Louis, MO) and 1% penicillin-streptomycin (Hyclone SV30010, Cytiva, Marlborough, MA). HCC1937 and 4T1 were obtained from the American Type Culture Collection (ATCC), and HCC1937-BRCA^29^ was kindly provided by Dr. S. Stecklein (University of Kansas Medical Center). The 4T1, HCC1937, and HCC1937-BRCA1 cells were cultured in RPMI-1640 medium with 10% heat-inactivated FBS (HyClone SH30910.03, Cytiva) and 1% penicillin-streptomycin. All cell lines were authenticated and tested negative for mycoplasma contamination.

### In-vitro irradiation conditions

**Fig. 7:**
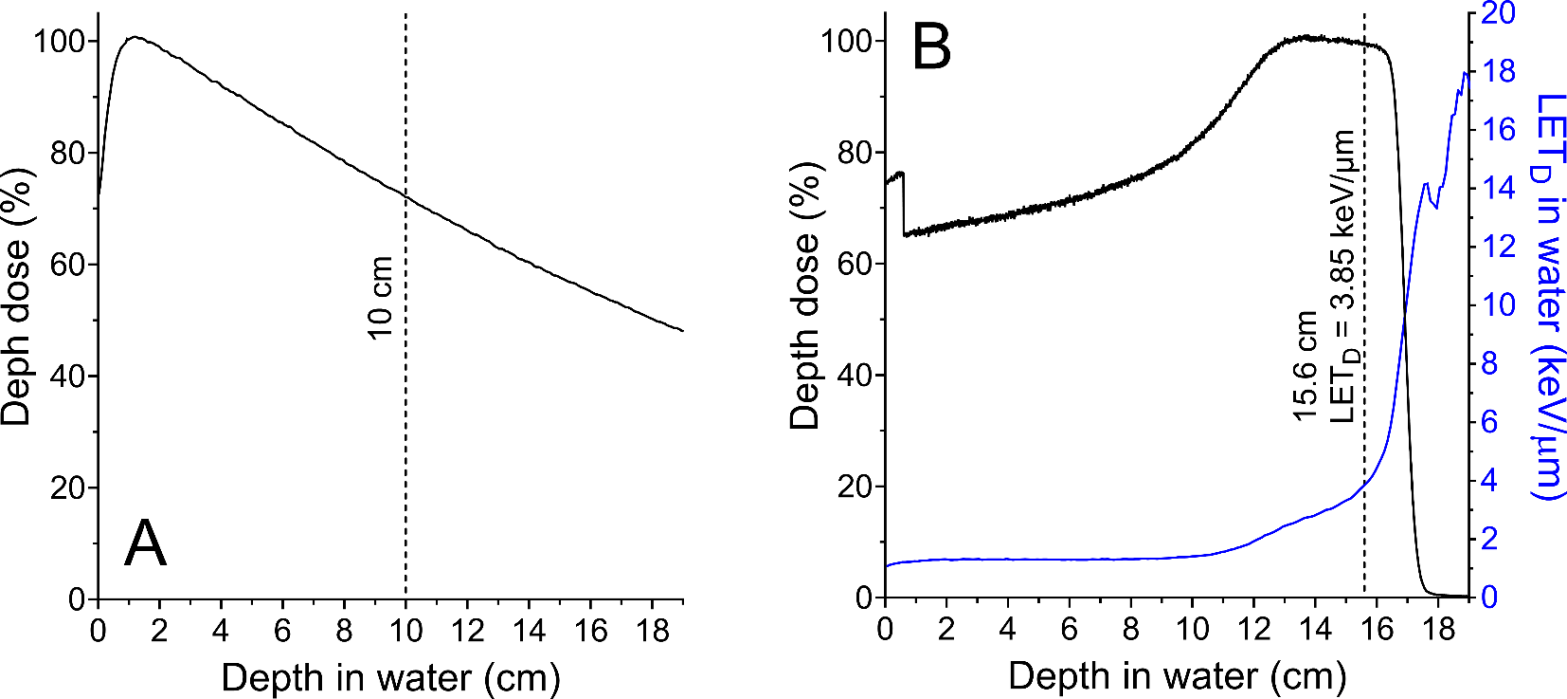
Physical characteristic of the photon and proton beams used for in vitro irradiations. (**A**) Percentage depth dose normalized at the maximum dose for the photon beam used to expose cells. (**B**) Percentage depth dose normalized at 15.6 cm depth and corresponding dose weighted LET in water as a function of depth for the proton beam used to expose cells. The proton depth dose and LET_D_ was calculated with a validated Monte Carlo model of the beam line. Dashed lines indicate the depth in which cells were exposed.

Photon irradiations were performed at the Proton Therapy Center in Houston, The University of Texas MD Anderson Cancer Center in parallel with the proton irradiations so that cells were subjected to same conditions, except the radiation type. Photon irradiations were done with 6 MV x-rays (Truebeam, Varian Medical System), field size 30 cm × 30 cm, gantry at 180° (beam from bottom to top) with flattening filter at a dose rate of 4.4 Gy/min. Before reaching the cells, the beam crossed the couch, water equivalent blocks and bottom of plates to a total water equivalent thickness (WET) of 10 cm. Plastic blocks (> 10 cm) were stacked upstream the plates to provide backscatter (Fig. 7A).

Proton irradiations were done with a double scattering nozzle using two different conditions. Most of the in vitro experiments were done with an unmodulated proton beam with a range (r_90_) in water of 4.3 cm at a water equivalent depth of 4.42 cm, resulting in a dose-weighted LET in water of 9.9 keV/µm, which was obtained with a validated Monte Carlo model of the proton beam nozzle^57^. Details of this irradiation setup can be found elsewhere^35^. Briefly, we used the range modulator wheel (RMW) 15 parked on the scatter foil (unmodulated beam), 18 cm × 18 cm field-size (medium snout fully retracted), 180° gantry angle at source-to-surface distance (SSD) setup to the bottom of the couch (beam from bottom to top). The beam crossed the couch, water-equivalent plastic blocks, and bottom of the 6-well plates to a combined water equivalent depth of 4.42 cm before reaching the cells. The proton dose rate ranged from 1 to 3 Gy/min, depending on the stability of the beam. The other condition used a modulated proton beam with a spread out Bragg peak (SOBP) of 4 cm and range (r_90_) in water of 16.5 cm (with the use of range shifters) at a water equivalent depth of 15.6 cm, resulting in a dose-weighted LET in water of 3.85 keV/µm, which was obtained with a validated Monte Carlo model of the proton beam nozzle^57^. We used the RMW 93, 18 cm × 18 cm field-size (medium snout fully retracted), gantry at 180° at SSD setup to the bottom of the couch (beam from bottom to top). Before reaching the cells, the beam crossed the couch, water equivalent blocks and bottom of plates to a total WET of 15.6 cm. The cells were positioned 1.1 cm distal to the middle of the SOBP position (Fig. 7B). The proton dose rate ranged from 1 to 3 Gy/min, depending on the stability of the beam.

### PARP inhibition

The PARP inhibitor Olaparib (AZD2281, S1060, Selleckchem, Houston, TX) was dissolved in dimethyl sulfoxide (DMSO) at 10 mM and was used at final concentrations of 0.1–10 µM. One hour before in vitro irradiation, medium was removed and replaced with medium containing either the inhibitor or DMSO vehicle. After 24 h of incubation, the medium was removed and replaced with fresh medium without PARP inhibitor or DMSO.

### Clonogenic assay

The cell lines were seeded 24 h before irradiation in T12.5 flasks (HCC1937 and HCC1937-BRCA1) or 6-well plates (MDA-MB-436, MDA-MB-436-BRCA1 and 4T1). Cells were then treated with DMSO or PARP inhibitor for 1 h, irradiated with photons or protons at doses ranging from 0 to 6 Gy, and stained at 9–18 days after irradiation. Colonies were stained with crystal violet (HT90132-1 L, Sigma Aldrich) diluted to 1:5 in absolute ethanol. Plates and flasks were air-dried overnight and then scanned with a high-resolution flatbed scanner (Expression 10000 XL, Epson, Los Alamitos, CA). Platting efficiencies were 13% for HCC1937; 10% for HCC1937-BRCA; 18.5% for MDA-MB-436, 21.6% for MDA-MB-436-BRCA and 53.0% for 4T1. Images were analyzed, and colonies with more than 50 cells were counted. The survival curves were then fit with the linear quadratic model to extract parameters of the cell survival curve such as D_10%_, the dose to reduce survival to 10%. We also defined the sensitization enhancement ratio (SER) at D_10%_ as the D_10%_ from survival curves in which cells were treated with DMSO divided by D_10%_ from survival curves in which cells were treated with PARP inhibitor. The relative biological effectiveness (RBE) at D_10%_ was defined as the D_10%_ for photons divided by the D_10%_ for protons for a given drug treatment, i.e., DMSO or PARP inhibitor.

### Micronuclei and DNA damage foci

Cells were plated in 96-well coverglass bottom plates (Sensoplate Microplate 655892, Greiner Bio-one, Monroe, NC) for 4 days before irradiation. At 24–72 h after irradiation, cells were fixed with 4% paraformaldehyde for 10 min at room temperature, washed with phosphate-buffered saline (PBS), and permeabilized with 0.3% Triton X-100 for 15 min at room temperature. Cells were then washed twice with PBS and incubated with blocking buffer consisting of 5% goat serum (AB7481, Abcam, Waltham, MA), 0.2% fish gelatin (G7041, Sigma-Aldrich), and 0.1% Tween 20 (P1379, Sigma Aldrich) in PBS for 1 h at room temperature. For HCC1937 and HCC1937-BRCA1 cells, primary antibodies to γH2AX (05-636, Millipore Sigma, Burlington, MA) and cGAS (15102 S, Cell Signaling Technology, Danvers, MA) were added and incubated overnight at 4 °C, after which the cells were washed three times and secondary antibodies (Alexa fluor 488 [A-11029, Thermo Fisher Scientific] and Alexa Fluor Plus 555 [A32732, Invitrogen]) were added and incubated for 1 h at room temperature. Images were acquired with a Cytation 5 Cell Imaging system (Agilent Technologies Inc., Santa Clara, CA). Foci, MN, and cGAS-positive MN cells were counted manually with ImageJ as follows. First, we manually counted the number of foci per nucleus for at least 500 cells in the control condition (0 Gy + DMSO). We then calculated a threshold defined by the mean foci number plus 2 × SD of the control condition to score foci in the treatment groups. Numbers of MN per nucleus were manually counted for at least 600 nuclei in each treatment group. The number of nuclei per image, number of MN-positive nuclei, and number of cGAS-positive MN were manually counted. Nuclei were counted as MN^+^ or cGAS^+^ if they had at least one associated micronucleus or cGAS-positive micronucleus, respectively.

### Cell cycle analysis

Cells were plated in 6-well plates at 4 days before irradiation so that the cells were subconfluent at irradiation. At 48 h after irradiation, cells were trypsinized and centrifuged for 5 min at 400×**g**, after which pellets were resuspended in 1 mL PBS with Ca^2+^ and Mg^2+^ (PBS+/+, 21-030-CV, Corning) with 5% FBS. Cells were then fixed by adding 2 mL of 70% ethanol drop-by-drop during gentle vortexing and stored at − 20 °C. After that, cells were washed with PBS without Ca^2+^ and Mg^2+^ (PBS−/−, SH30256.01, HyClone) with 2% FBS and spun down for 5 min at 400×*g*. PBS-/- and 2% FBS were then added to cells for 15 min at room temperature for blocking. Then, propidium iodide (PI) (FxCycleTM PI/RNase Staining Solution, F10797, Invitrogen) was added for 15 min at room temperature. Flow cytometry measurements were obtained with an Attune NxT Acoustic Focusing Cytometer (Invitrogen, Thermo Fisher Scientific, Waltham, MA). Flow cytometry data were analyzed with FlowJo 10.7.1 software (FlowJo, LLC, Ashland, OR).

### Senescence (C12FDG)

Because senescent cells are known to overexpress β-galactosidase, we assessed senescence as described by Debacq-Chainaux et al.^30^. Briefly, at 7 days after irradiation, cells were pretreated for 1 h with bafilomycin A1 (100 nM, S1413, Selleckchem) and then for 2 h with 5-dodecanoylaminofluorescein di-B-D-galactopyranoside (C_12_FDG), which fluoresces upon cleavage by β-galactosidase (33 µM, D2893, Invitrogen), at 37 °C with 5% CO_2_. Cells were trypsinized and centrifuged at 4 °C at 400×*g* for 5 min. Pellets were resuspended in 1 mL of cold PBS+/+ with 5% heat inactivated FBS. Living cells were selected before flow cytometry by adding the cell viability marker To-Pro-3 (T3605, Invitrogen) to the samples, which were then assessed with an Attune NxT Acoustic Focusing Cytometer. Data were analyzed with FlowJo 10.7.1 software.

### Animal experiments

Tumor implantation, irradiation, and drug treatment procedures were done in accordance with an animal use protocol. This protocol was approved by our Institutional Animal Care and Use Committee (IACUC) (1590-RN02). Methods are reported in accordance with ARRIVE guidelines. BALB/c mice were purchase from Envigo (Indianapolis, IN) and housed (2–5 per cage) in a pathogen-free facility with standard controlled temperature (72 °F), humidity (30–70%), and a light cycle of 12 h on/12 h off set from 7 am to 7 pm. Mice had unrestricted access to standard food and water under the supervision of veterinarians. Mice that reached our euthanasia criteria were humanely euthanized by CO_2_ inhalation as per IACUC guidelines. All efforts were made to minimize animal suffering including administration of isoflurane for anesthesia as required. 4T1 was inoculated subcutaneously in the left leg of female BALB/c mice. Only female mice were used in this study because breast cancer is prevalent in this sex. Seven days after inoculation, tumors were treated with photons alone, photons + PARP inhibitor (Olaparib), protons alone, or protons + PARP inhibitor. The PARP inhibitor was administered by oral gavage on three consecutive days at a dose of 100 mg/kg per day. The first dose of PARP inhibitor was given 2 h before the tumor was irradiated with a single 11-Gy dose as follows.

**Fig. 8:**
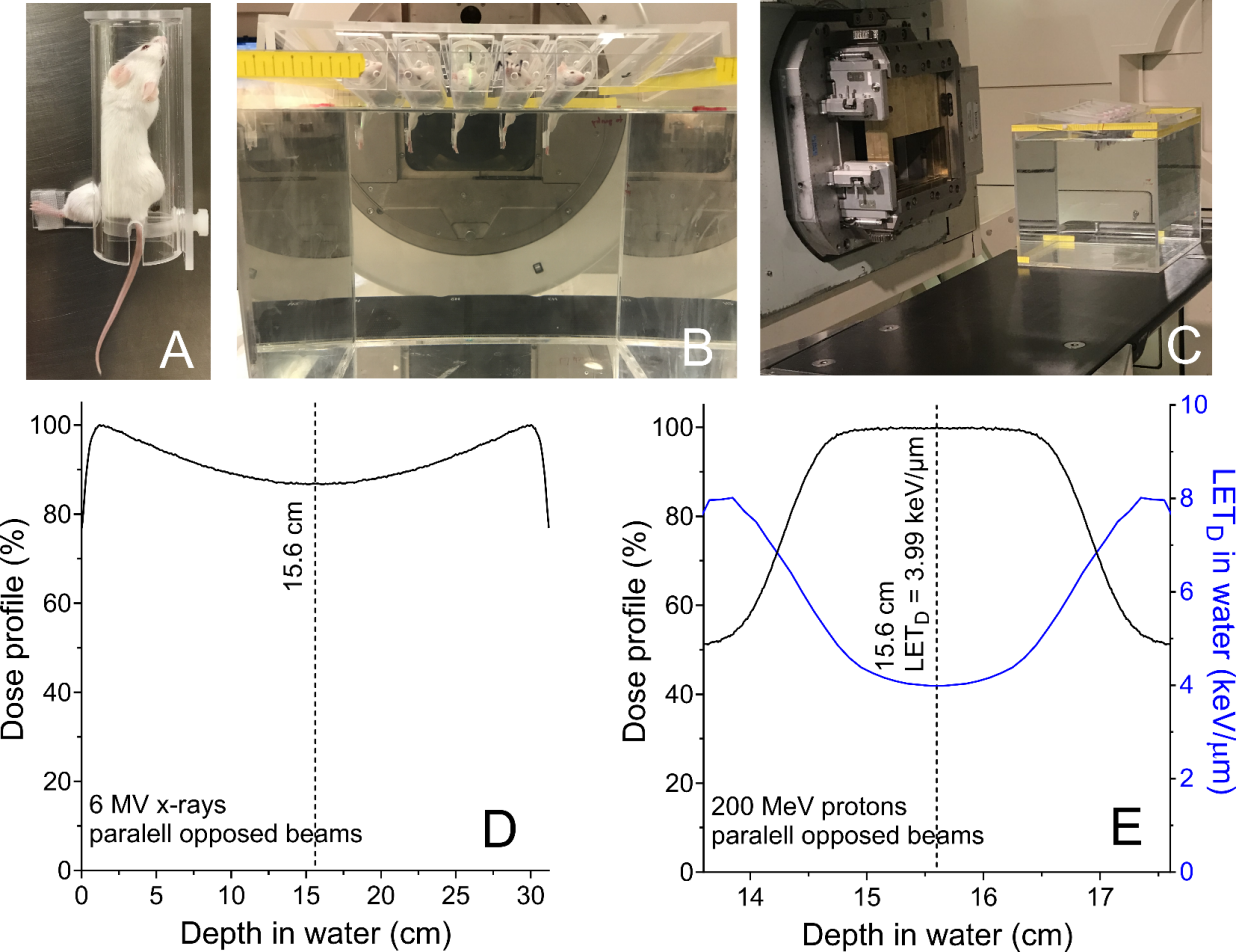
Irradiation setup and physical characteristic of the photon and proton beams used for in vivo irradiations. (**A**) Restrainer used to immobilize the left leg. (**B**,**C**) Custom-designed jig in a water tank. The jig holds up to six mice, with their left legs (and tumors) inside a water tank. A half-beam block is used to spare the mouse’s body and internal organs from radiation. This setup allows each individual tumor to be precisely aligned to the radiation field. (**D**) Dose profile in the water tank used for photon (6 MV x-rays) irradiations with opposite lateral beams. (**E**) Dose profile and corresponding dose-weighted LET (LET_D_) profile in the water tank used for proton irradiations with lateral opposed beams. The proton dose profile and LET_D_ was calculated with a validated Monte Carlo model of the beam line. Dashed lines represent the depth in which the tumors were exposed.

Mice were immobilized with custom-built restrainers that isolate the left (tumor-bearing) legs and then placed in a jig that allowed the isolated and extended leg to be immersed in a water tank (31.2 cm length with 0.6 cm Lucite walls) so that clinical radiation beams could be delivered at a fixed depth with high precision and throughput (Fig. 8A–C).

Photon irradiations were performed at the Proton Therapy Center in parallel with the proton irradiations so that animals were subjected to same conditions, except the radiation type. Photon irradiations were done with 6 MV x-rays (Truebeam), field size 30 cm × 30 cm, gantry at 0° and 270° with a half-beam block. Parallel opposed beams were used for the irradiations to create a homogenous dose distribution. A water tank (31.2 cm length with 0.6 cm Lucite walls) located with center (15.6 cm) at isocenter (ISO) was used. The physical distance from the upstream phantom surface to ISO was 15.6 cm (Fig. 8D).

Proton irradiations were done with a double scattering nozzle using the RMW 26 (option 11), field Size 25 cm × 25 cm (large snout fully retracted), range (*r*_90_) = 16.5 cm, SOBP = 4 cm, gantry at 0° and 270°. Parallel opposed beams were used for the irradiations to create homogenous dose and LET distributions (3.99 keV/µm). We used the same water tank and setup that we used for the irradiations with photons. (Fig. 8E).

### Statistical analysis

Statistical analyses were performed with GraphPad Prism (GraphPad Software, San Diego, CA). All data with more than two groups were analyzed by one-way ANOVA with Tukey post hoc test. Two-tailed unpaired t-test was used to compare two groups. A 95% of confidence level was used. Time for tumor volumes to reach a threshold value was estimated by the Kaplan–Meier method, and curves were compared with log-rank (Mantel-Cox) tests (GraphPad Prism). The threshold used to consider a statistical difference was *p* < 0.05.

## Electronic supplementary material

Below is the link to the electronic supplementary material.

Supplementary Material 1

## Data Availability

All data generated or analyzed during this study are included in this published article (and its Supplementary Information file).
