# Supplementary material for: PARP inhibition radiosensitizes BRCA1 wildtype and mutated breast cancer to proton therapy
Source: Sci Rep. 2024 Dec 28;14:30897. doi: 10.1038/s41598-024-81914-w (PMC11680706; doi:10.1038/s41598-024-81914-w)
Supplement: Supplementary file 1 — Supplementary Material 1 [file 41598_2024_81914_MOESM1_ESM.pdf]

Supplementary Information

PARP inhibition radiosensitizes BRCA1 wildtype and mutated breast cancer to proton therapy

Mariam Ben Kacem, Scott J. Bright, Emma Moran, David B. Flint, David K. J. Martinus, Broderick X. Turner, Ilsa Qureshi, Rishab Kolachina, Mandira Manandhar[1], Poliana C. Marinello, Simona F. Shaitelman, and Gabriel O. Sawakuchi

Supplementary Data: Figs S1-S10 and Tables S1-S6

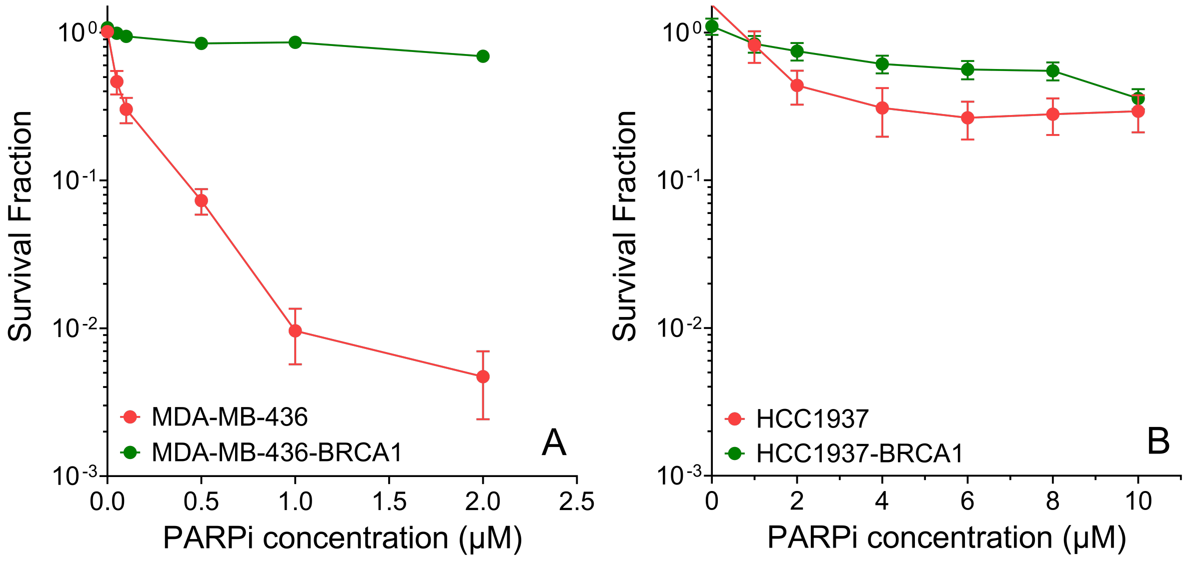

Figure S1. PARP inhibitor titration for MDA-MB-436 cells (A) and HCC1937 cells (B). Red indicates BRCA1 mutated cells, and green BRCA1 restored cells.

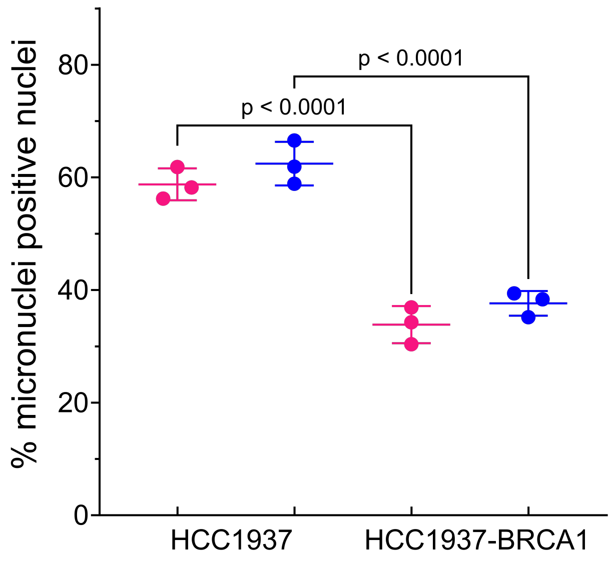

Figure S2. Micronuclei in HCC1937 (BRCA1 mutated) and HCC1937-BRCA1 (BRCA1 restored) cells at 72 h after irradiation with photons (6 MV x-rays) or protons (9.9 keV/μm) with DMSO. Error bars represent standard deviation. Each bar represents the mean of at least 3 independent experiments. One-way ANOVA with Tukey post hoc test was used to estimate p values.

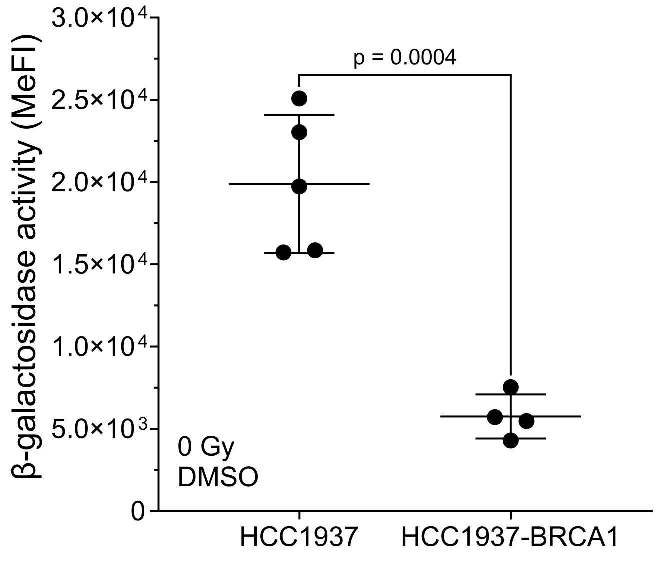

Figure S3. SA-β-galactosidase levels for HCC1937 (BRCA1 mutated) and HCC1937-BRCA1 (BRCA1 restored) cells treated with dimethylsulfoxide (DMSO) only. Each bar represents the mean of at least four independent experiments for HCC1937 and HCC1937-BRCA1. Error bars represent standard deviation. MeFI, mean fluorescence intensity. Unpaired t test was use to calculate the p value.

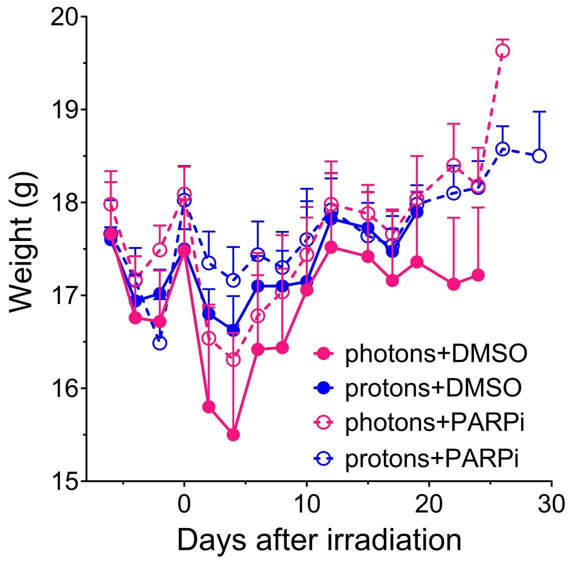

Figure S4. Animal weight as a function of time after irradiation with photons or protons plus vehicle (DMSO) or PARPi (3 consecutive days at a dose of 100 mg/kg per day starting 2 h before irradiation) administered by oral gavage. Mice were inoculated with 4T1 cells on day -7 and were exposed to a single 11-Gy dose of photons (6 MV x-rays) or protons (9.9 keV/μm) on day 0. Error bars represent the standard error of the mean.

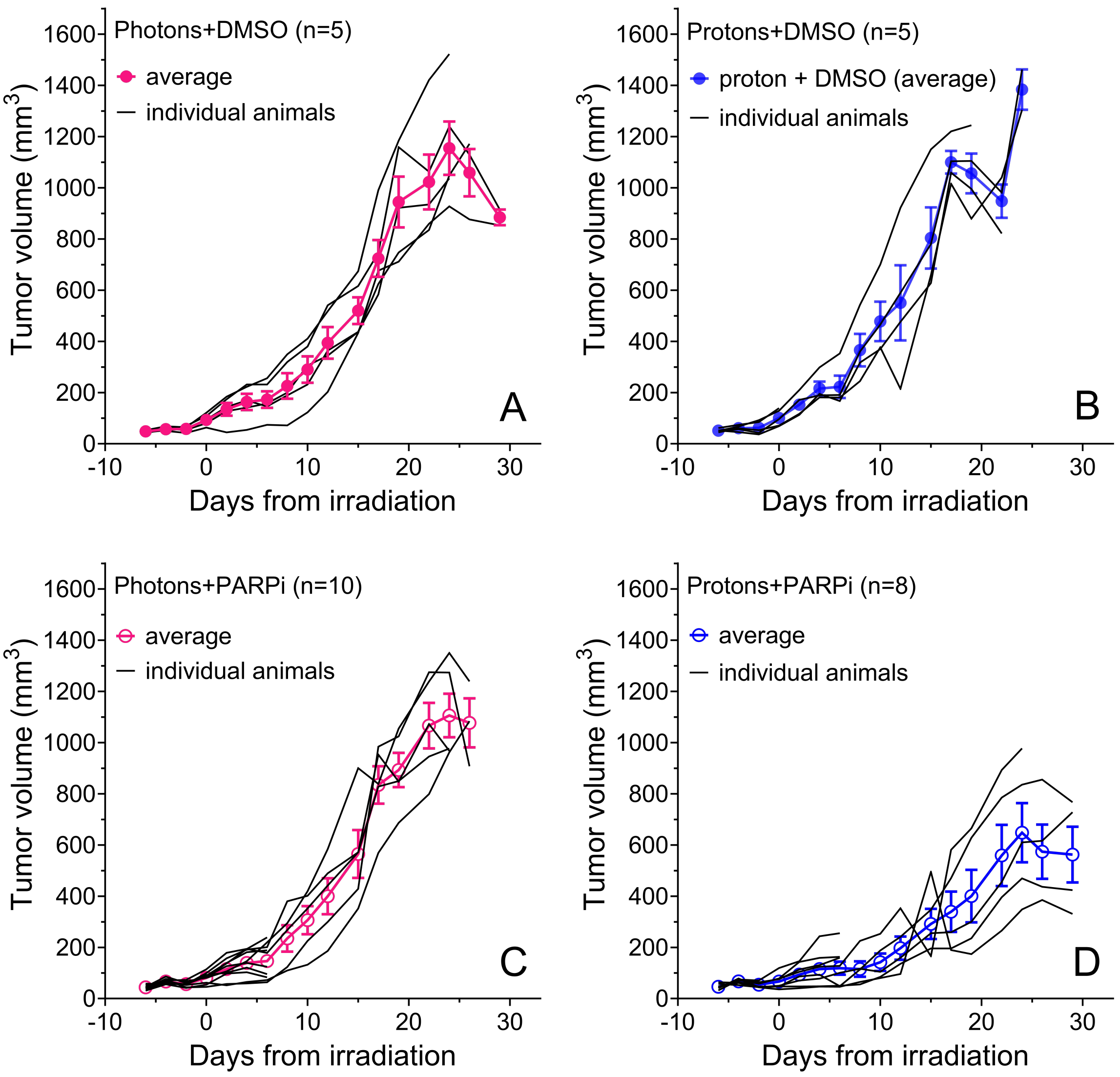

Figure S5. Individual tumor growth delay for mice with photons+DMSO (A), protons+DMSO (B), photons+PARPi (C) and protons+PARPi (D) treatments. Mice were inoculated with 4T1 cells on day -7 and were exposed to a single 11-Gy dose of photons (6 MV x-rays) or protons (9.9 keV/μm) on day 0. Error bars represent standard deviation.

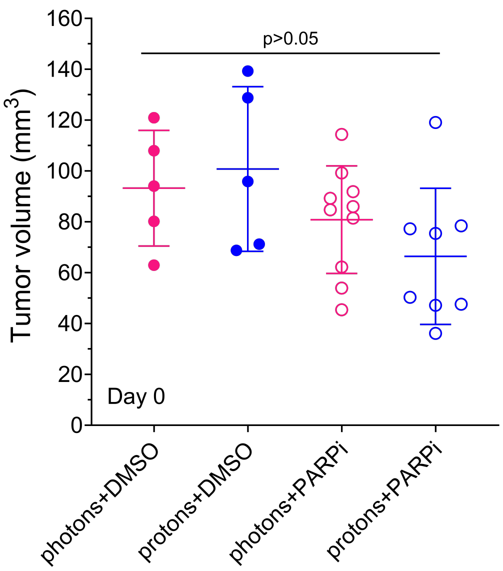

Figure S6. Tumor volume at Day 0 for all animals per group of treatment. Mice were inoculated with 4T1 cells on day -7 and were exposed to a single 11-Gy dose of photons (6 MV x-rays) or protons (9.9 keV/μm) on day 0. Error bars represent standard deviation. One-way ANOVA with Tukey post hoc test was used to estimate p values.

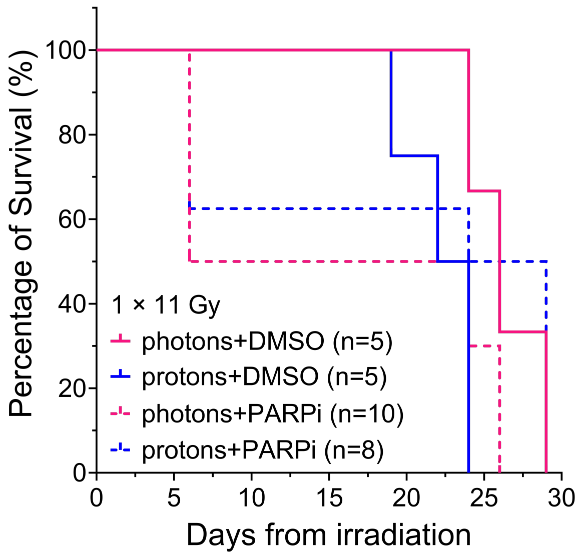

Figure S7. Overall survival in mice as a function of time after irradiation with photons or protons plus vehicle or PARPi (3 consecutive days at a dose of 100 mg/kg per day starting 2 h before irradiation) administered by oral gavage. Survival curves were compared with log-rank (Mantel-Cox) tests (GraphPad Prism). Mice were inoculated with 4T1 cells on day -7 and were exposed to a single 11-Gy dose of photons (6 MV x-rays) or protons (9.9 keV/μm) on day 0.

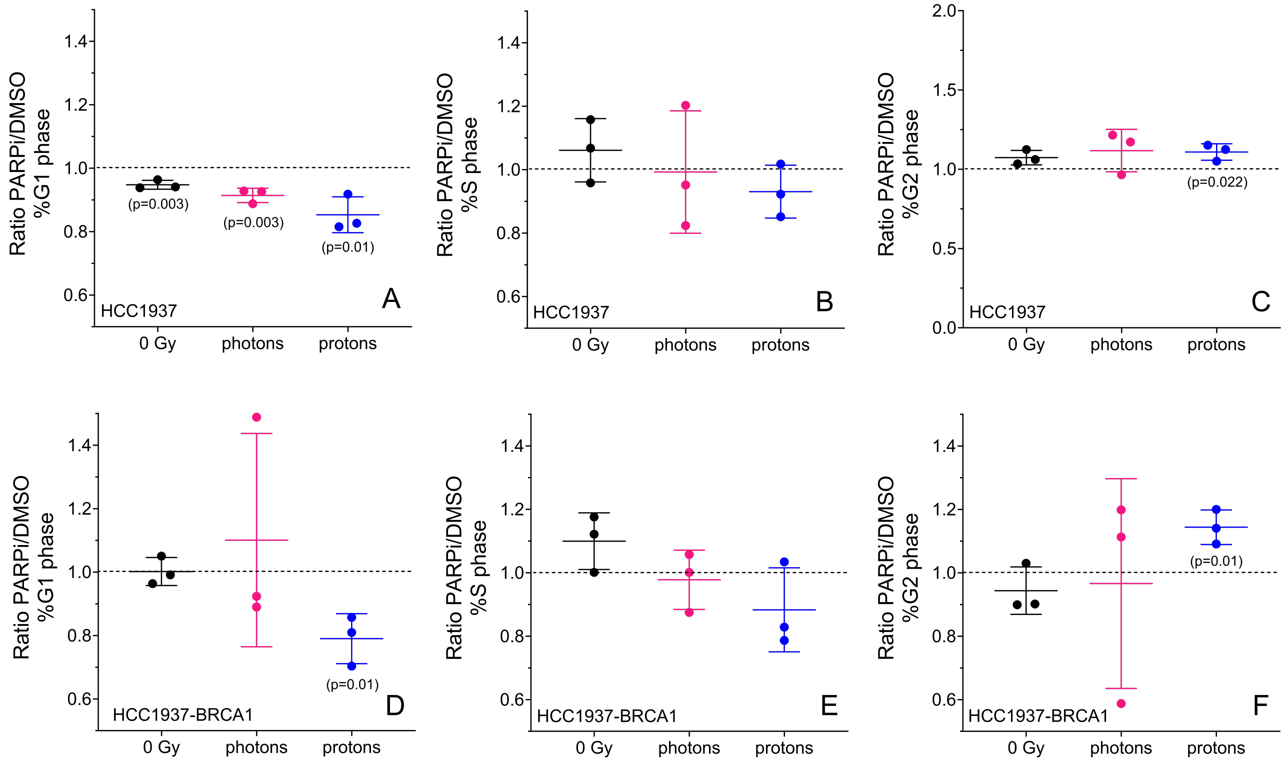

Figure S8. Ratio of cell percentage at each cell cycle phase after radiation. Each condition represents the mean of three independent experiments. Error bars represent standard deviation. An unpaired t test was used to compare ratios with a reference of 1 (p value indicated in parenthesis).

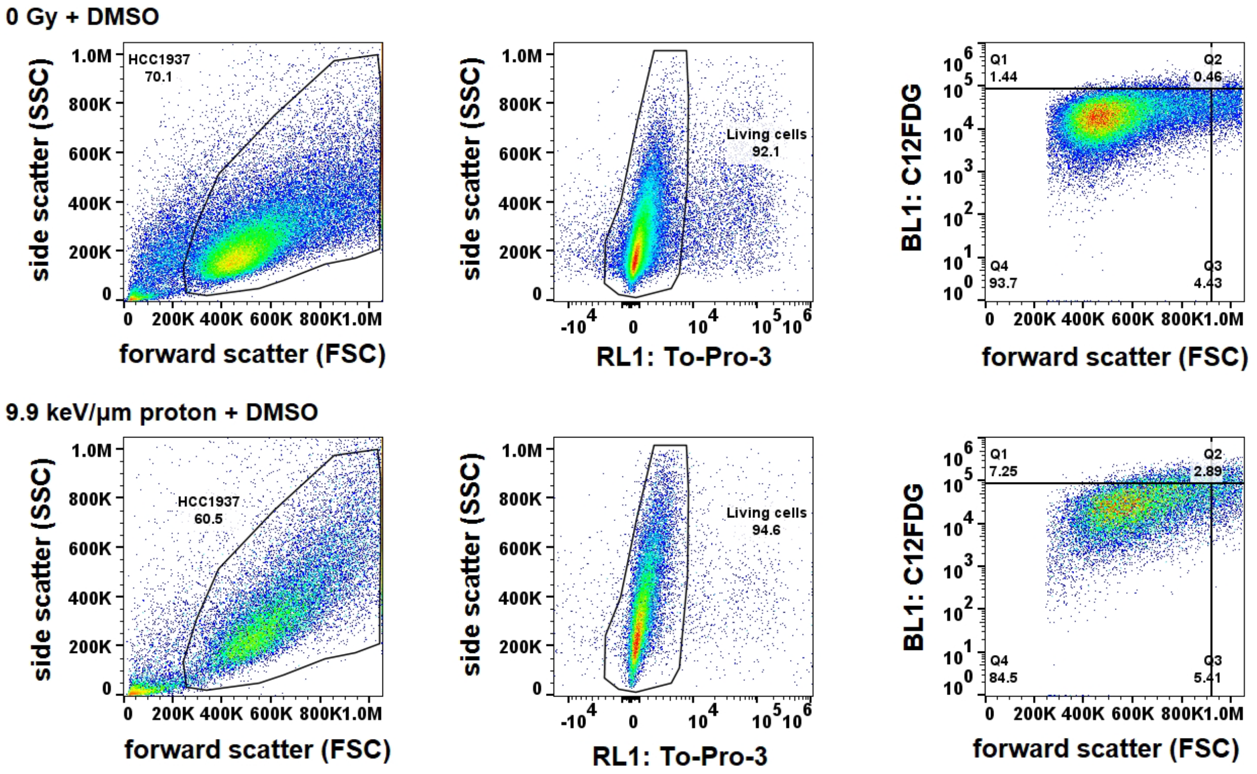

Figure S9. Stress-induced senescence with gating to determine β-galactosidase activity levels.

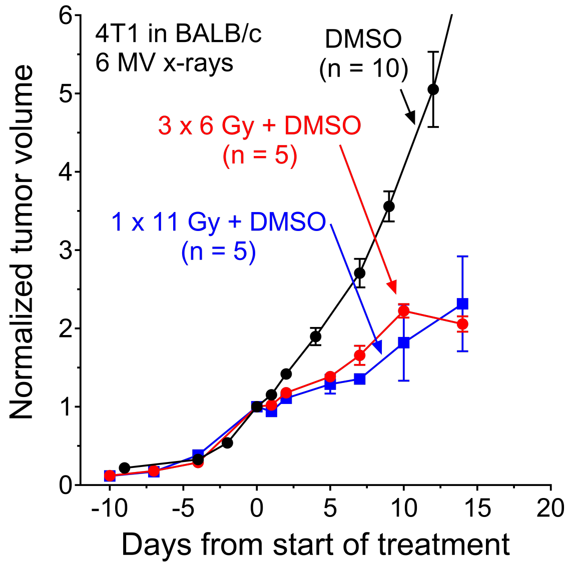

Figure S10. Comparison of tumor growth delay using either one fraction of 11 Gy or three fractions of 6 Gy spaced 18-24 h apart to a total of 18 Gy. The data was normalized by the tumor volume on the day of the first fraction.

Supplementary Tables S1-S6

Table S1. $D_{10\%}$ (dose to reduce survival to 10%) values (with SDs), their standard deviation and number of repeats from clonogenic assays of the four human breast cancer cell lines HCC1937, HCC1937-BRCA, MDA-MB-436, and MDA-MB-436-BRCA; and one murine cell line 4T1.

	**Cell lines**				
	**HCC1937**	**HCC1937-BRCA1**	**MDA-MB-436**	**MDA-MB-436-BRCA1**	**4T1**
**Treatment Condition**			***$D_{10\%}$, Gy***		
Photon + DMSO	2.51±0.16 (n=3)	1.88±0.26 (n=5)	4.90±0.19 (n=4)	6.25±0.18 (n=3)	7.35±0.04 (n=7)
Proton + DMSO	1.65±0.14 (n=3)	1.35±0.10 (n=5)	3.07±0.11 (n=4)	3.73±0.08 (n=3)	6.31±0.13 (n=6)
Photon + PARPi	1.67±0.10 (n=3)	1.30±0.13 (n=5)	3.17±0.14 (n=3)	4.55±0.21 (n=3)	5.44±0.04 (n=4)
Proton + PARPi	1.39±0.08 (n=3)	0.65±0.08 (n=5)	2.53±0.10 (n=3)	3.38±0.11 (n=3)	4.38±0.03 (n=3)

Table S2. SER at $D_{10\%}$ (sensitization enhancement ratio) values (with SDs) from clonogenic assays of the four human breast cancer cell lines HCC1937, HCC1937-BRCA, MDA-MB-436, and MDA-MB-436-BRCA; and one murine cell line 4T1.

	**Cell lines**				
	**HCC1937**	**HCC1937-BRCA1**	**MDA-MB-436**	**MDA-MB-436-BRCA1**	**4T1**
**Treatment Condition**			***SER at $D_{10\%}$***		
Photon + DMSO	1<br>(n=3)	1<br>(n=5)	1<br>(n=4)	1<br>(n=3)	1<br>(n=7)
Proton + DMSO	1<br>(n=3)	1<br>(n=5)	1<br>(n=4)	1<br>(n=3)	1<br>(n=6)
Photon + PARPi	1.50±0.13<br>(n=3)	1.44±0.25<br>(n=5)	1.54±0.09<br>(n=3)	1.38±0.07<br>(n=3)	1.352±0.013<br>(n=4)
Proton + PARPi	1.19±0.13<br>(n=3)	2.09±0.31<br>(n=5)	1.21±0.06<br>(n=3)	1.10±0.04<br>(n=3)	1.44±0.03<br>(n=3)

Table S3. RBE at $D_{10\%}$ values (with SDs) from clonogenic assays of the four human breast cancer cell lines HCC1937, HCC1937-BRCA, MDA-MB-436, and MDA-MB-436-BRCA; and one murine cell line 4T1.

	**Cell lines**				
	**HCC1937**	**HCC1937-BRCA1**	**MDA-MB-436**	**MDA-MB-436-BRCA1**	**4T1**
**Treatment Condition**	***RBE at $D_{10\%}$***				
DMSO	1.52±0.16 (n=3)	1.39±0.22 (n=5)	1.59±0.08 (n=4)	1.68±0.06 (n=3)	1.166±0.025 (n=6)
PARPi	1.20±0.10 (n=3)	2.01±0.33 (n=5)	1.25±0.0.08 (n=3)	1.35±0.08 (n=3)	1.243±0.013 (n=3)

Table S4. α values (with SDs) for the four human breast cancer cell lines HCC1937, HCC1937-BRCA, MDA-MB-436, and MDA-MB-436-BRCA; and one murine cell line 4T1.

	**Cell lines**				
	**HCC1937**	**HCC1937-BRCA1**	**MDA-MB-436**	**MDA-MB-436-BRCA1**	**4T1**
**Treatment Condition**			***α, Gy^{-1}***		
Photon + DMSO	0.69±0.17 (n=3)	1.38±0.18 (n=5)	0.19±0.04 (n=4)	0.21±0.03 (n=3)	0.059±0.012 (n=7)
Proton + DMSO	1.23±0.30 (n=3)	1.70±0.13 (n=5)	0.32±0.10 (n=4)	0.42±0.04 (n=3)	0.18±0.05 (n=6)
Photon + PARPi	0.96±0.25 (n=3)	1.77±0.18 (n=5)	0.38±0.11 (n=3)	0.26±0.09 (n=3)	0.106±0.019 (n=4)
Proton + PARPi	1.66±0.1 (n=3)	3.56±0.45 (n=5)	0.76±0.12 (n=3)	0.34±0.08 (n=3)	0.240±0.017 (n=3)

Table S5. β values (with SDs) for the four human breast cancer cell lines HCC1937, HCC1937-BRCA, MDA-MB-436, and MDA-MB-436-BRCA; and one murine cell line 4T1.

	**Cell lines**				
	**HCC1937**	**HCC1937-BRCA1**	**MDA-MB-436**	**MDA-MB-436-BRCA1**	**4T1**
**Treatment Condition**			***β, Gy^{-2}***		
Photon + DMSO	0.093±0.08<br>0 (n=3)	0<br>(n=5)	0.058±0.013<br>(n=4)	0.026±0.007<br>(n=3)	0.0345±0.0017<br>(n=7)
Proton + DMSO	0.10±0.19<br>(n=3)	0<br>(n=5)	0.14±0.03<br>(n=4)	0.054±0.011<br>(n=3)	0.029±0.008<br>(n=6)
Photon + PARPi	0.25±0.16<br>(n=3)	40<br>(n=5)	0.11±0.04<br>(n=3)	0.054±0.020<br>(n=3)	0.058±0.003<br>(n=4)
Proton + PARPi	0<br>(n=3)	0<br>(n=5)	0.059±0.049<br>(n=3)	0.10±0.02<br>(n=3)	0.065±0.003<br>(n=3)

Table S6. α/β ratio values (with SDs) for the four human breast cancer cell lines HCC1937, HCC1937-BRCA, MDA-MB-436, and MDA-MB-436-BRCA; and one murine cell line 4T1.

	Cell lines				
	**HCC1937**	**HCC1937-BRCA1**	**MDA-MB-436**	**MDA-MB-436-BRCA1**	**4T1**
**Treatment Condition**	*α/β ratio, Gy*				
Photon + DMSO	7.4±6.6 (n=3)	* (n=5)	3.2±1.0 (n=4)	7.9±2.4 (n=3)	1.72±0.34 (n=7)
Proton + DMSO	12.2±23.0 (n=3)	* (n=5)	2.24±0.87 (n=4)	7.7±1.8 (n=3)	6.13±2.41 (n=6)
Photon + PARPi	3.8±2.6 (n=3)	* (n=5)	3.5±1.7 (n=3)	4.9±2.4 (n=3)	1.81±0.35 (n=4)
Proton + PARPi	* (n=3)	* (n=5)	13.0±11.1 (n=3)	3.3±1.1 (n=3)	3.68±0.30 (n=3)

***** Alpha/beta ratios could not be calculated for HCC1937 and HCC1937-BRCA1 cell lines as their beta values are 0.
